# Diversification and Population Structure in Common Beans (*Phaseolus vulgaris* L.)

**DOI:** 10.1371/journal.pone.0049488

**Published:** 2012-11-07

**Authors:** Matthew W. Blair, Alvaro Soler, Andrés J. Cortés

**Affiliations:** 1 Departamento de Ciencias Agrícolas, Universidad Nacional de Colombia, Palmira, Colombia; 2 Department of Plant Breeding, Cornell University, Ithaca, New York, United States of America; 3 Evolutionary Biology Center, Uppsala University, Uppsala, Sweden; New York State Museum, United States of America

## Abstract

Wild accessions of crops and landraces are valuable genetic resources for plant breeding and for conserving alleles and gene combinations *in planta*. The primary genepool of cultivated common beans includes wild accessions of *Phaseolus vulgaris.* These are of the same species as the domesticates and therefore are easily crossable with cultivated accessions. Molecular marker assessment of wild beans and landraces is important for the proper utilization and conservation of these important genetic resources. The goal of this research was to evaluate a collection of wild beans with fluorescent microsatellite or simple sequence repeat markers and to determine the population structure in combination with cultivated beans of all known races. Marker diversity in terms of average number of alleles per marker was high (13) for the combination of 36 markers and 104 wild genotypes that was similar to the average of 14 alleles per marker found for the 606 cultivated genotypes. Diversity in wild beans appears to be somewhat higher than in cultivated beans on a per genotype basis. Five populations or genepools were identified in structure analysis of the wild beans corresponding to segments of the geographical range, including Mesoamerican (Mexican), Guatemalan, Colombian, Ecuadorian-northern Peruvian and Andean (Argentina, Bolivia and Southern Peru). The combined analysis of wild and cultivated accessions showed that the first and last of these genepools were related to the cultivated genepools of the same names and the penultimate was found to be distinct but not ancestral to the others. The Guatemalan genepool was very novel and perhaps related to cultivars of race Guatemala, while the Colombian population was also distinct. Results suggest geographic isolation, founder effects or natural selection could have created the different semi-discrete populations of wild beans and that multiple domestications and introgression were involved in creating the diversity of cultivated beans.

## Introduction

Common bean (*Phaseolus vulgaris* L.) is a diverse New World legume species that originated in a long arc between present day northern Mexico (Chihuahua), through Central America and the Andes mountains to northwest Argentina (San Luis) [1]. The diversity in wild accessions of the species can be divided into various sub-populations from specific geographical regions for the species [2], [3]. The number of sub-populations has been a matter of discussion since the division of wild *P. vulgaris* is not as simple as for the domesticated beans which are easily separated into Andean and Mesoamerican genepools. In addition, the morphological and molecular differences among groups of wild accessions are not as clear as among races in the cultivated types and rely on differences in seed size, flower coloration, bracteoles size, seed protein (phaseolin) type and in large part on molecular marker evaluations [2], [4], [5], [6], [7], [8]. It is uncertain when the full transition from wild beans to cultivated beans occurred due to gaps in the archaeological record but this event is thought to have occurred 7,000 to 5,000 years ago [1].

Based on DNA fingerprinting with amplified fragment length polymorphism (AFLP) markers, wild common bean accessions have been divided into four groups or genepools [9]. These include Mesoamerican, Andean, Colombian and Ecuadorian-northern Peruvian genepools. Some studies with Andean wild and cultivated common beans with the same marker system found no grouping of wild accessions within the Andean genepool [10]. However, northern Argentinean and southern wild Bolivian accessions have been suggested to be most similar to cultivated Andean beans. Rossi *et al.* [5] also found geographic separation of wild bean populations with AFLP markers suggesting that Colombian wild beans were closely related to Mesoamerican wild beans which could be separated into accessions from Mexico and Central America. They also suggested a reduction in diversity in the Andean genepool. Kwak *et al.* [11] found that wild beans from Mexico varied in their simple sequence repeat (SSR) fingerprint and that domestication of the cultivated Mesoamerican genepool was likely to have occurred in the Lerma valley. Introgression of wild-derived genes from other subgroups of wild beans has been postulated to explain current SSR-based race structure in cultivated common beans [12], [13], [14]. Finally based on sequence information for five gene fragments, Bitocchi *et al.* [15] proposed a Mesoamerican origin rather than a South American origin for wild populations of *P. vulgaris* based on the detection of a strong bottleneck in the actual Andean genepool of wild beans.

Microsatellite or SSR markers are useful tools for studying genetic diversity in multiple crops and their wild relatives [16]. This type of marker, based generally on di- or tri-nucleotide repeats, is highly polymorphic and multi-allelic, with up to 25 alleles common at an individual locus. Microsatellite loci are abundant and well distributed throughout the genomes of higher plants, being found in both gene-coding and non-coding sequences. SSR markers are easily evaluated through fluorescent marker technology [14]. In addition, data from microsatellite analysis can be reproducible from laboratory to laboratory and robust for comparisons between studies and germplasm sets. The application of microsatellite markers to study diversity within common beans began when Gaitán *et al.* [17] developed a set of genomic microsatellites and evaluated their diversity in cultivated and wild accessions of common beans and related species. Metais *et al.* [18] also evaluated diversity for other genomic microsatellites and fluorescently labeled SSR markers. A more comprehensive analysis of the diversity of cultivated accessions was then carried out by Blair *et al.* [19] to evaluate which microsatellites best detected diversity within the species. Subsequently race structure was analyzed in cultivated Andean and Mesoamerican beans [12], [13] with some of the most reliable markers from the previous study. Later, Kwak and Gepts [4] evaluated 349 cultivated and wild accessions with 26 markers to make inferences about population structure in the species. For the most part, their results for cultivated beans agreed with a simultaneous analysis from Blair *et al.* [14] where 604 cultivated genotypes from a core collection were evaluated with a standardized set of 36 microsatellite markers.

The main objective of this research was to evaluate the number of sub-populations in wild common beans and to relate these to races of cultivated common beans. The specific goals of this study were 1) to evaluate a large set of over one hundred wild common bean accessions with the same large panel of microsatellite markers as in Blair *et al.* [14], 2) to combine the analysis of wild accessions described here with that previous analysis of cultivated landraces, and 3) to determine the population structure and the ongoing processes of differentiation for wild versus cultivated beans. The wild accessions were from a wild bean core collection representative of the geographic range of the species and were morphologically and genetically diverse. The markers used were the same set of fluorescent microsatellites evaluated by Blair *et al.* [14] allowing the combined analysis of results from wild common beans used in this study and cultivars used in the previous study. This is the largest number of wild and cultivated accessions of common bean to have been analyzed with a standard genotyping protocol.

## Materials and Methods

### Plant Materials

A total of 108 genotypes were used in the fingerprinting analysis of this study. Of these 108, 88 were wild, 16 were weedy and four were cultivated check genotypes used in previous studies from our laboratory [14], [19]. All the genotypes were from the Genetic Resources Unit at the International Center for Tropical Agriculture and are preserved under the treaty for genetic resources from the Food and Agriculture Organization, hereafter abbreviated as the FAO collection. The set of accessions conform a core collection for wild *P. vulgaris* as described in Tohme *et al.* [9]. This core collection was based on ecological classification of the geographical origin of each accession which is found at http://isa.ciat.cgiar.org/urg/main.do.

The wild and weedy genotypes were from the following countries arranged from north to south: Mexico (45), El Salvador (1), Guatemala (11), Costa Rica (1), Colombia (11), Ecuador (5), Peru (17), Bolivia (3) and Argentina (10). Meanwhile, the four check genotypes represented the Mesoamerican (Dorado and ICA Pijao) and Andean genepools (Calima and Chaucha Chuga), with germplasm entries DOR364, G5773, G19833 and G4494 also from the FAO collection. For each wild accession three seeds were scarified by hand by cutting through the seed coat opposite the micropyle with a razor blade prior to planting in sterilized soil in a 10 inch diameter pot in a screen-house. Multiple plants were used to determine if the accessions were heterozygous and to obtain sufficient leaf tissue for DNA extraction since wild beans have small leaves. The accessions had been previously selected for homogenous seed shape, color and size.

### DNA Extraction and Microsatellite Analysis

Leaf tissue weighing approximately 20 mg was harvested at 35 days after plant germination and freeze dried in a MODULYoD-115-Thermo® liophylizer for two days after which it was ground to a fine powder with a ceramic mortar and pestle. Freeze drying was found to be more appropriate than grinding in liquid N2 for wild bean leaf tissue which is rich in carbohydrates and tannins compared to cultivated bean leaf tissues. The ground tissue was then used for DNA extraction in a 2 mL eppendorf tube with the ingredients from a Viogene DNA kit. DNA was quantified in 1% agarose gels using Ethidium bromide staining and Quantity One® v 4.0.3 evaluation of the resulting GelDoc 2000 (Bio-Rad®) images comparing lanes for each DNA extraction with lanes representing 25, 50, 100, 200 and 400 ng/ul concentrations of λ phage DNA. Uniform DNA concentrations of 5 ng/ul were then used for PCR reactions.

A total of 36 fluorescently-labeled microsatellites as listed in Blair *et al.* (2009) were employed to evaluate the wild accessions and cultivated checks. These included 17 gene-based markers and 19 genomic markers distributed in nine panels of four markers each. For each marker within each panel, the forward primers were 5′ end labeled with one of the following fluorochromes: 6-FAM, NED, PET or TET. Microsatellites were amplified on PTC-200 thermocyclers (MJ-Research) in 15 uL reaction volumes using 20 ng of template DNA, 3 pmole of each primer, 1.5 mM of MgCl_2_ [c5], 0.6 mM of dNTP and 1 U of *Taq* polymerase in 1 X PCR buffer (10 mM of Tris–HCl pH 8.8, 50 mM of KCl, 0.1% of TritonX-100).

The thermocyling profile was the following: 95°C hot start for 3 min, followed by 28 cycles of 95°C denaturation for 40 s, 55°C annealing for 40 s and 72°C extension for 1 min with a 1-h extension at 72°C was used post-thermocycling. The resulting PCR products were evaluated for thermocyling reaction efficiency on 1.5% agarose gels and then diluted and combined into panels as *per* Blair *et al.* [14]. The LIZ500 size standard was diluted into formamide and was then added to the mixed PCR products and these were denatured at 94°C for three minutes. The denatured sample was then loaded onto an ABI 3730xl automated sequencer (Applied Biosystem, Foster City, CA) at the Institute for Genomic Diversity of Cornell University.

### Data Analysis for Wild Beans

Band or alleles sizes were estimated in base-pairs with GeneMapper v. 3.7 software (Applied Biosystems). Allele binning was conducted with AlleloBin software (http://www.icrisat.org/gt-bt/biometrics.htm) which groups band sizes based on the algorithm of Idury and Cardon [20]. Whole-integer, binned allelic data was used to calculate genetic dissimilarity based on the proportion of shared alleles in Darwin v.5 software (Perrier *et al.* 2003).

Meanwhile the software PowerMarker [21], was used to determine the number of polymorphic alleles (Na), the genetic diversity index of Nei [22] and the expected heterozygosity and polymorphic information content (PIC) for each marker [23]. In addition, the genetic distance matrix was used to construct a dendogram in Darwin v.5 software using the neighbor-joining algorithm [22].

Population structure was first examined with STRUCTURE software [24], which determines a *Q* matrix of population relatedness and tests the K value of possible sub-populations found in a sample of genetic diversity. A total of 15 independent runs were used for each K value from K = 2 to K = 10 using an admixture model and 100,000 replicates both in the burn-in and MCMC analysis. A bar graph of the population structure results was generated for each K value using Distruct software [25] that was labeled with the drawing software PowerPoint™ 2010 (Microsoft Office).

Following this method, a second population structure analysis was performed using InStruct software [26]. A correlation model for allele frequency was performed using 100,000 burn-ins and 200,000 iterations in InStruct. Permutations of the output of STRUCTURE and Instruct analysis were performed with CLUMPP software [27] using independent runs to obtain a consensus matrix based on 15 simulations. The final structure of the population was determined based on the germplasm information, cross-run cluster stability, and likelihood of the graph model from Evano *et al.* [28].

In addition to analyzing population structure, the genetic relationship among all accessions was analyzed in three dimensions by principal coordinates analysis using the program Genalex [29] and plotted through XLSTAT-3D ™ program (http://www.xlstat.com/es/home/) using Microsoft™ Office Excel 2010. The geographical distribution of wild accessions was visualized with the program DIVA-GIS [30]. Genalex [29] was used to perform a Mantel’s test to estimate the correlation between the matrices of genetic distance and geographic distance, the latter drawn from latitude and longitude.

In the Mantel’s test the genetic distance matrix was based on the proportion of shared alleles as calculated with Darwin software and the geographic distance matrix was calculated with DIVA-GIS. Finally, an analysis of molecular variance (AMOVA) was performed to assess the differentiation among subpopulations using Arlequin v. 3.11 [31].

### Data Analysis for Global Diversity Set

The analysis of the wild accessions described above was combined with a previous analysis of cultivated landraces by merging the present dataset with the dataset of Blair *et al.* (2009) (Supplemental Table S1). The correspondence between alleles from different sets was carefully checked and several inconsistent markers (BMd01, BM205) were excluded. The analyses described for the wild accessions in the previous paragraphs were repeated using the integrated matrix. The number of chains for the burn-in and for the estimation of the posterior distribution in the STRUCTURE analysis was triplicated, though. This guaranteed convergence and consistency across all independent runs from K = 2 to K = 16 so that we could determine the most a parsimonious scenario to understand how diversity was structured across wild and cultivated common beans. K-level divisions were based on assignments of genotypes to wild bean sub-populations as described above or to cultivated races as described in [14]. The naming convention of the wild sub-populations was based on the STRUCTURE and PCoA analysis results and the previously recognized genepools for wild beans in Broughton *et al.* (2003), while the naming convention for cultivated races was based on those proposed by Blair *et al.* (2009).

## Results

### Allelic Diversity of the Wild Accessions

A total of 492 alleles were detected in the wild bean core collection using the 36 fluorescent markers. This resulted in an average of 13 alleles per marker. All the markers except for BMd51 detected polymorphism (Table 1). The average PIC value was 0.64 and the average expected heterozygosity was 0.66. PV-at001 was the marker with the highest expected heterozygosity (0.96) which was to be predicted since this gene-based marker presented a total of 40 different alleles. The genomic markers GATs91 and BM143 also had high expected heterozygosities (above 0.93). On average the genomic microsatellites had a higher number of alleles (17), higher expected heterozygosity (0.77) and PIC values (0.75) compared to gene-based microsatellites (with values of 9 alleles, 0.55 H_e_ and 0.51 PIC, respectively). Non-amplification (null alleles) was only a problem for BM140 and BM187 with all other markers having from 85 to 100% of the expected data points. On average marker amplifications provided 96% of the expected data points.

**Table 1:** Genetic diversity parameters for 36 microsatellite markers evaluated on 104 wild common bean accessions.

SSR locus	Number of Alleles	Expected heterozygosity	Observed heterozygosity	PIC
**Genomic**				
AG01	7	0.70	0.02	0.66
BM137	12	0.30	0.02	0.30
BM139	15	0.74	0.00	0.70
BM140	20	0.81	0.09	0.80
BM141	22	0.87	0.13	0.85
BM143	22	0.93	0.11	0.93
BM149	6	0.36	0.09	0.34
BM156	26	0.88	0.17	0.87
BM160	17	0.85	0.02	0.83
BM172	17	0.78	0.04	0.75
BM175	21	0.92	0.12	0.91
BM183	17	0.89	0.18	0.88
BM187	25	0.90	0.15	0.89
BM188-A	8	0.60	0.09	0.56
BM188-B	20	0.92	0.00	0.92
BM200	22	0.92	0.12	0.91
BM201	16	0.68	0.39	0.67
BM205	13	0.81	0.12	0.79
GATs54	14	0.61	0.05	0.59
GATs91	24	0.94	0.16	0.94
**Mean**	**17**	**0.77**	**0.10**	**0.75**
**Gene-based**				
BMd01	17	0.91	0.31	0.91
BMd02	5	0.46	0.08	0.43
BMd08	7	0.56	0.06	0.53
BMd15	9	0.37	0.16	0.35
BMd16	4	0.50	0.01	0.39
BMd17	6	0.35	0.01	0.33
BMd18	7	0.80	0.09	0.77
BMd20	7	0.63	0.05	0.59
BMd46	5	0.59	0.05	0.50
BMd47	5	0.49	0.02	0.41
BMd51	1	0.00	0.00	0.00
BMd56	3	0.49	0.02	0.42
PV-ag003	6	0.46	0.01	0.43
PV-at001	40	0.96	0.09	0.96
PV-at003	9	0.63	0.11	0.58
PV-cct001	5	0.40	0.10	0.36
PV-ctt001	12	0.82	0.15	0.80
**Mean**	**9**	**0.55**	**0.08**	**0.51**
**Total Mean**	**13**	**0.66**	**0.09**	**0.64**
**TOTAL**	**492**			

Observed heterozygosity for the markers was 0.09 on average but was highest for the markers BMd01 and BM201 which were multiple banding and difficult for allele calling compared to the other markers. Some other markers had observed heterozygosity values between 0.13 and 0.18 even though they were easily read as single-copy bands. These included BM141, BM183, BM187 and GATs91 among the genomic markers or BMd15 and PV-ctt001 among the gene-based markers. Differences between the genomic and gene-based microsatellites for observed heterozygosity were not significant as these had similar averages of 0.10 and 0.08, respectively.

### Population Structure and Dendogram of the Wild Accessions

Evaluation of population structure in the wild beans using K = 2 to K = 10 sub-populations resulted in similar separations for the two software programs used (STRUCTURE and Instruct) and therefore the first of these softwares is presented. The ideal K-value was selected based on the increases in likelihood ratios between runs using Evano’s delta K statistic [28]. Points of inflection were not observed for the log-likelihood curve but a smaller increase of the likelihood was found when comparing K = 5 to previous K-values (Supplemental Figure S1).

**Figure 1:**
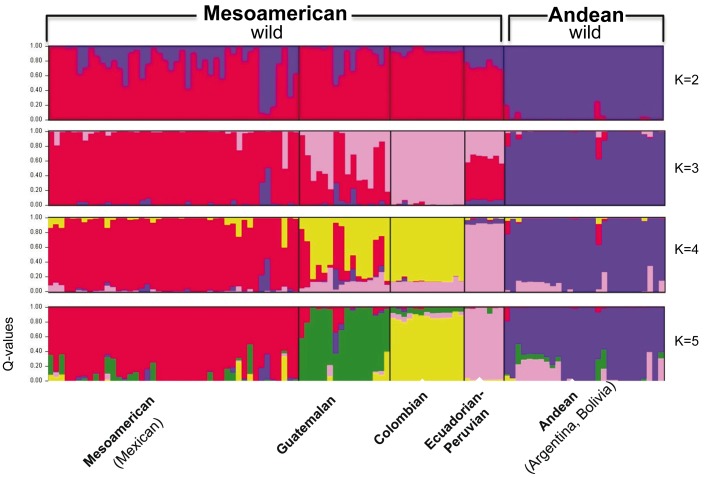
Population structure analysis for 104 wild accessions of common bean and 4 control genotypes based on microsatellite marker analysis. K-values of 2 to 5 sub-populations are shown to right and naming of wild common bean genepools given below and assignment to Andean or Mesoamerican groups shown above.

Separation of the sub-populations at each K-value in STRUCTURE was instructive and is presented in Figure 1. At the first level of sub-population separation, K = 2, the wild genotypes divided into two genepools roughly of Andean and Mesoamerican types. At K = 3 the Colombian genotypes separated from the other two genepools. At K = 5 the sub-population separation agreed with geographical distribution along latitudinal demes of Mesoamerican (Mexican), Guatemalan, Colombian, Ecuadorian-northern Peruvian and Andean (Argentinean, Bolivian and Southern Peru) populations. We favored K = 5 because a second peak was found for Evano test values at this K-value (Supplemental Figure S1). The first K value was for K = 2 which represented the Andean – Mesoamerican split.

**Figure 2:**
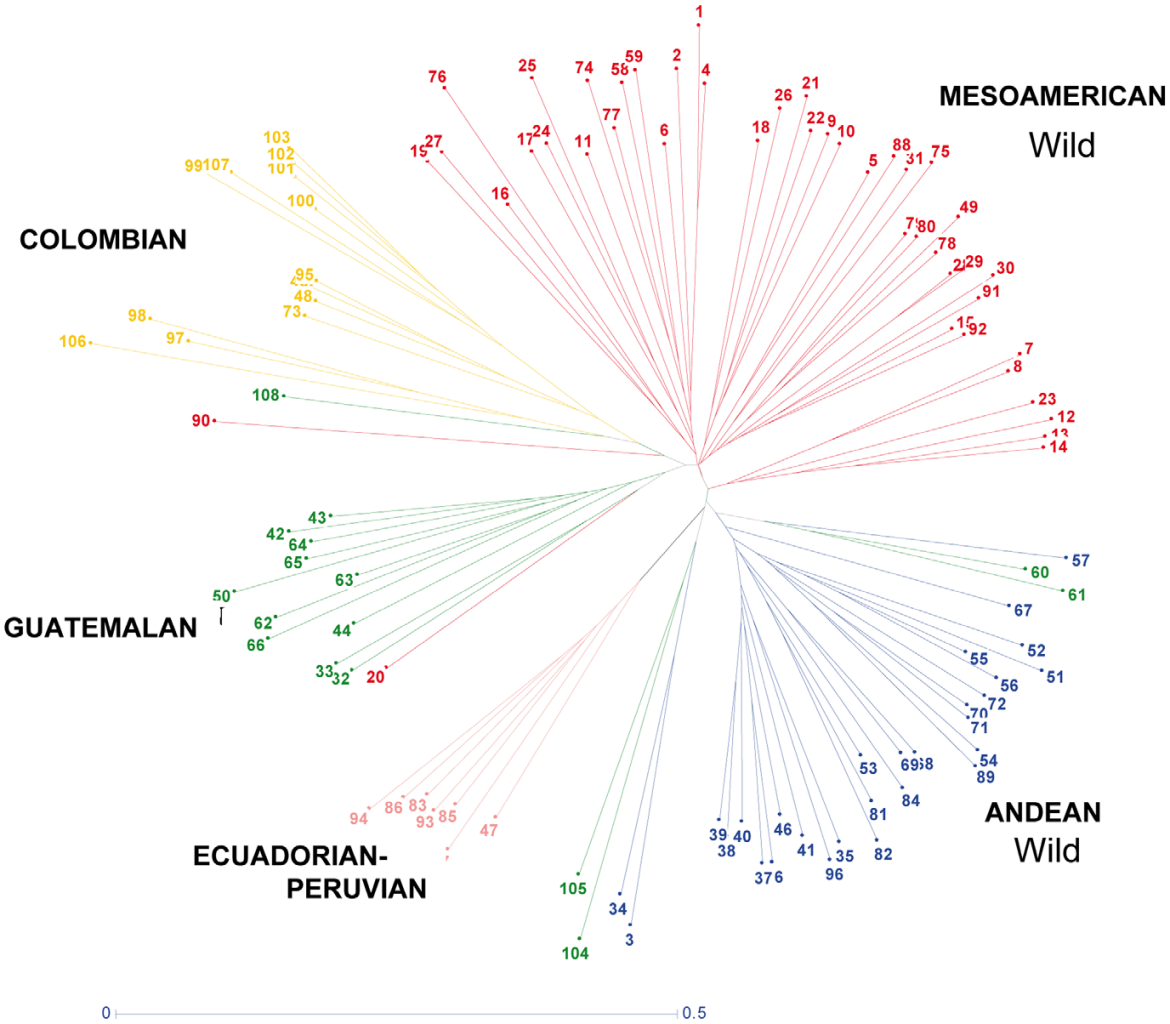
Neighbor-joining dendogram of wild accessions of common beans with sub-populations based on population structure analysis shown in previous figure.

The Neighbor Joining dendogram constructed with the dissimilarity matrix for the wild genotypes (Figure 2) corroborated the assignment of genotypes to sub-populations in STRUCTURE, with good separation of most of the wild accession genepools. Ecuadorian-Peruvian genotypes all grouped together in the NJ dendrogram while Colombian and Gautemalan genepools were more similar to specific Mesoamerican accessions. Andean accessions did not all group together but rather separated into three purely Andean groups and one admixed group (both with Andean and Guatemalan sub-population accessions).

### Geographical Analysis of Wild Populations

**Figure 3:**
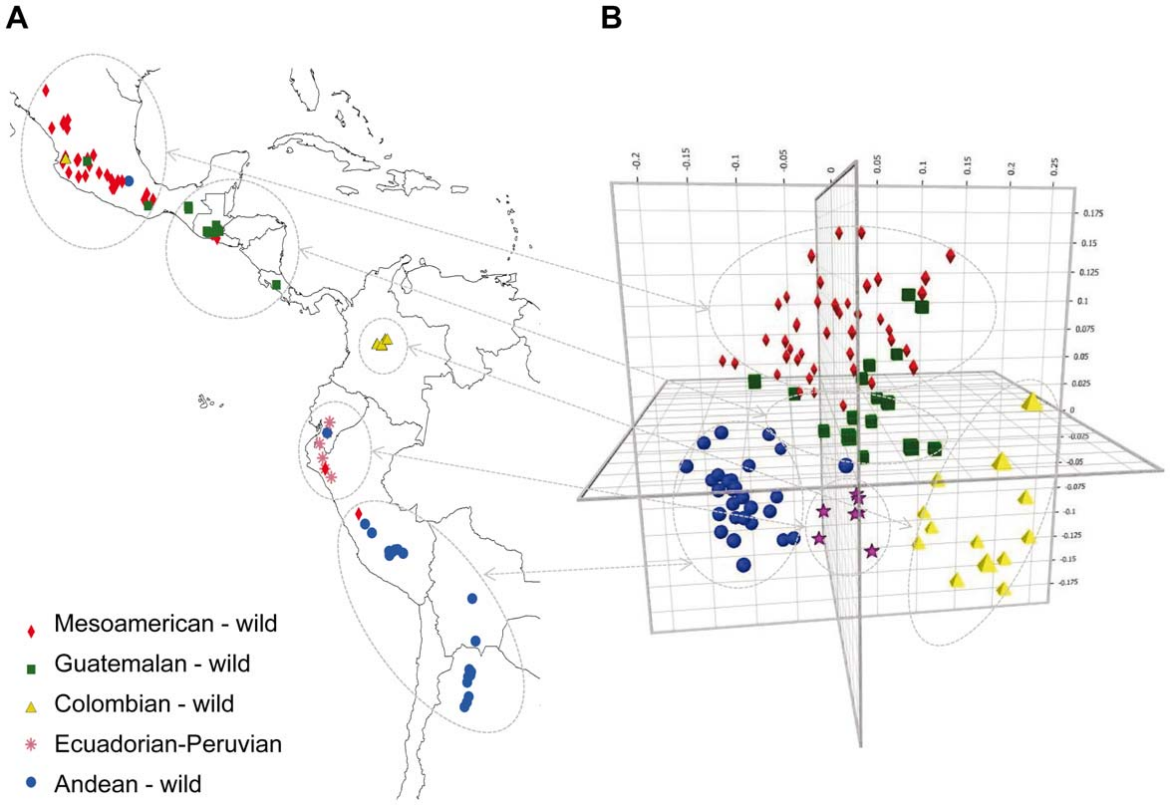
Geographical distribution of the collections sites for wild bean accessions genotyped in this study (A) and their assignment by principal component analysis to five genepools based on population structure analysis with microsatellite markers (B). Each genepool is shown with a different symbol/color.

Figure 3A shows the geographic distribution of wild accessions based on information on collection site and their genepool assignment in STRUCTURE. Admixed individuals were assigned to the genepool for which Q was greater than 0.5. A clear geographic separation of the sub-populations was found based on the source region for each wild genepool. For example Guatemalan genepool of wild beans came almost exclusively from Guatemalan and Mexican highlands. The states of Mexico that contributed wild beans to the Guatemalan sub-population were Chiapas, Jalisco and Oaxaca. One additional wild bean from a medium elevation site in Costa Rica was included in this subgroup of wild common beans.

The PCoA analysis carried out for the wild accessions (Figure 3B) confirmed the sub-populations described above and showed their relationships in three dimensional space. The percentages of genetic diversity explained by each of the three coordinates of the PCoA were 28.5%, 20.9% and 14.9% for the first, second and third dimensions, respectively. The full analysis separated the Andean, Colombian, Guatemalan, Mesoamerican and Ecuadorian-northern Peruvian wild bean sub-populations into clusters shown with different colored symbols in Figure 3B. In this analysis, almost all of the Mesoamerican wild beans came from Mexico (88.1%) with a few accessions from El Salvador and lowland Guatemala (2.4% each). The Colombian sub-population included mostly wild beans from Colombia (84.6%) but also a few Mexican wild beans which would have to be confirmed as sharing ancestry with the Colombian wild beans. Among the Andean genepool accessions, the wild beans were mostly from Argentina (34.6%), Bolivia (11.5%) and Peru (46.2%). The last sub-population of wild beans consisted entirely of accessions from Ecuador and Northern Peru but overlapped in the geographical range with the most northerly originating accessions of the Andean sub-population.

A Mantel’s test for correlation between the matrices of genetic distances (genetic dissimilarity based on the proportion of shared alleles) and geographic distances (uncorrected Euclidean distance between collection points for any two accessions) was significant and positive (r = 0.193, P<0.010), suggesting an association of population structure and isolation of genotypes by latitudinal-longitudinal distance. The overall analysis of geographical spread of the wild bean accessions shows that representation of the species range was good with the full range from northern Mexico to northern Argentina covered in this study. The only geographic gaps in the distribution of the wild accessions would be in southern Colombia, central Peru and a few parts of Mesoamerica (Panama for example) where it has been difficult to collect beans due to inaccessibility and political upheavals or where very few wild beans exist in the first place due to the inhospitable climate or ecology of the region (cloud or rainforest areas).

### Genetic Differentiation of Sub-populations

An analysis of molecular variance (AMOVA) resulted in significant variation between the five wild bean genepools (P<0.0001) with 20.3% of variation being attributable to population differences (Supplemental Table S2). Genetic differentiation between sub-populations showed low values (F_st_≤0.08) between the Colombian and Guatemalan genepools, and between the Mesoamerican genepool and these previous genepools (Table 2). Similarly low values of genetic differentiation were found for Andean versus Colombian or Guatemalan genepools, indicating that these two genepools are intermediate between the Andean and Mesoamerican groups of wild beans. Meanwhile, levels of genetic differentiation were also fairly low (0.08≤F_st_≤0.10) for Andean versus Mesoamerican comparisons and were moderate (0.10≤F_st_≤0.20) for comparisons of Ecuadorian-northern Peruvian and Andean, Colombian or Guatemalan genepools. Therefore, the Ecuadorian-northern Peruvian genepool was the most distinct from the genepools of South and Central America. However, this Ecuadorian-northern Peruvian genepool was fairly similar to the Mesoamerican genepool (F_st_ = 0.067) showing that it might be related to this group of wild beans in North America.

**Table 2:** Genetic differentiation based on F_st_ values between five wild common bean genepools identified with population structure analysis after microsatellite genotyping of 104 accessions.

	MW	GW	CW	ENPW	AW
**Mesoamerican (MW)**	**–**				
**Guatemalan (GW)**	0.07509	**–**			
**Colombian (CW)**	0.07372	0.03305	**–**		
**Ecuadorian-Peruvian (ENPW)**	0.06686	0.14389	0.19120	**–**	
**Andean (AW)**	0.08225	0.06807	0.07027	0.13328	**–**

The F_st_ value for genetic differentiation between all the populations was 0.203 which is relatively high confirming the separation of all the wild genepools and their diversity in SSR alleles. In terms of variability within each wild bean genepool, diversity seemed to be greatest in the Mesoamerican genepool (H_e_ = 0.625) and slightly less in the Ecuadorian-northern Peruvian genepool (H_e_ = 0.430). The Andean genepool had intermediate values (0.507) as did the Colombian (0.602) and Guatemalan (0.594) genepools.

Observed heterozygosity values in the wild sub-populations varied from 0.061 to 0.111 and were correlated with expected heterozygosity. Average allele number was highest in the Mesoamerican genepool (8.1), followed in order by the Andean genepool (6.8), the Guatemalan genepool (5.2) and the Ecuadorian-northern Peruvian genepool (2.9). The number of alleles was proportional also to the number of individuals in each subpopulation (Table 3).

**Table 3:** Genetic diversity parameters for five wild common bean genepools identified with population structure analysis of microsatellite genotyping of 104 accessions.

Genepool	Number of individuals	Allele number	Exp. het.	Obs. het.	% poly.
Mesoamerican (MW)	44	8.135	0.625	0.111	97.3
Guatemalan (GW)	16	5.162	0.594	0.089	94.6
Colombian (CW)	13	4.622	0.602	0.087	97.3
Ecuadorian-N. Peruvian (ENPW)	7	2.865	0.430	0.061	67.6
Andean (AW)	28	6.784	0.507	0.071	94.6
**Total Wild collection**	**108**	**5.514**	**0.551**	**0.084**	**90.3**

### Comparison of Wild Accessions to Cultivated Genotypes

Upon combining the dataset for the wild common beans with the microsatellite screening of cultivated common beans using most of the same markers from Blair *et al.* (2009), we found that the combined analysis was informative both in terms of population structure using the software program STRUCTURE and for the global PCoA graph. In the combined analysis, 33 microsatellite loci were evaluated given that three markers (BMd01, BM188 and BM201) were multiple banding and were eliminated for the comparison.

**Figure 4:**
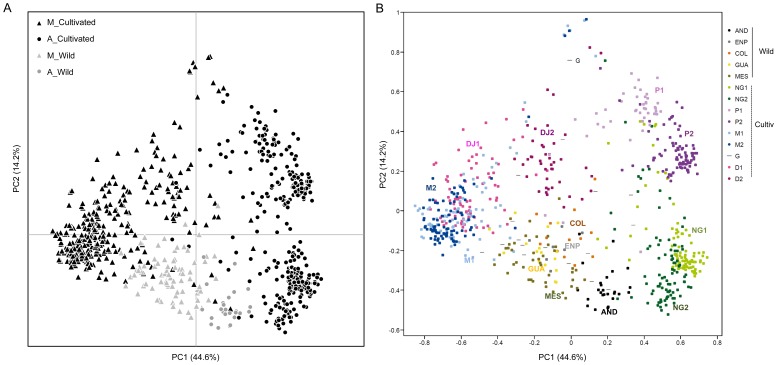
PCoA analysis of the associations between wild (W) common beans and races of cultivated (C) common beans with panel A showing only genepool and wild versus cultivated bean differences and panel B showing the same analysis but with aggregates of cultivated races and wild sub-populations as defined by the legend. A three letter code is used to name the wild populations, while the conventions of Blair *et al.* [14] were used for races: DJ: Durango-Jalisco, M: Mesoamerica, NG: Nueva-Granada, P: Peru, and G: Guatemala.

The PCoA analysis carried out for the integrated dataset (Figure 4A) confirmed the sub-populations described above for wild beans and the races described by Blair *et al.* [14] for the cultivated core collection. The percentages of genetic diversity explained by each of the two main coordinates of the PCoA were 44.6% and 14.9% for the first and second dimensions, respectively. Interestingly, wild beans occupied an intermediate, comparably narrow space between the cultivars. Nevertheless, the Andean wild genotypes were closer to the Andean cultivars than any of the other wild sub-populations (Colombian, Guatemalan and Mesoamerican-Mexican and Ecuadorian northern Peruvian), which were closer in turn to the cultivated Mesoamerican group (Figure 4B). The full analysis separated the Andean, Colombian, Ecuadorian Northern Peruvian, Guatemalan and Mesoamerican sub-populations, and the Nueva-Granada, Peru, Mesoamerica and Durango-Jalisco races as defined by Blair *et al.* (2009). Some intra-race subdivision was also detected, especially within the Andean races. Evano’s delta K favored K = 2 for the global analysis, corresponding to the Andean-Mesoamerican split. In order to explore deeper population structure, independent Structure analyses were carried out for each genepool. Evano’s delta K for the intra-genepool structure analyses favors K = 5 for both the Andean and Mesoamerican genepools (Supplemental Figure S1). We explored higher K-values for the Mesoamerican analysis taking into account *a priori* information such as races that have been previously reported.

**Figure 5:**
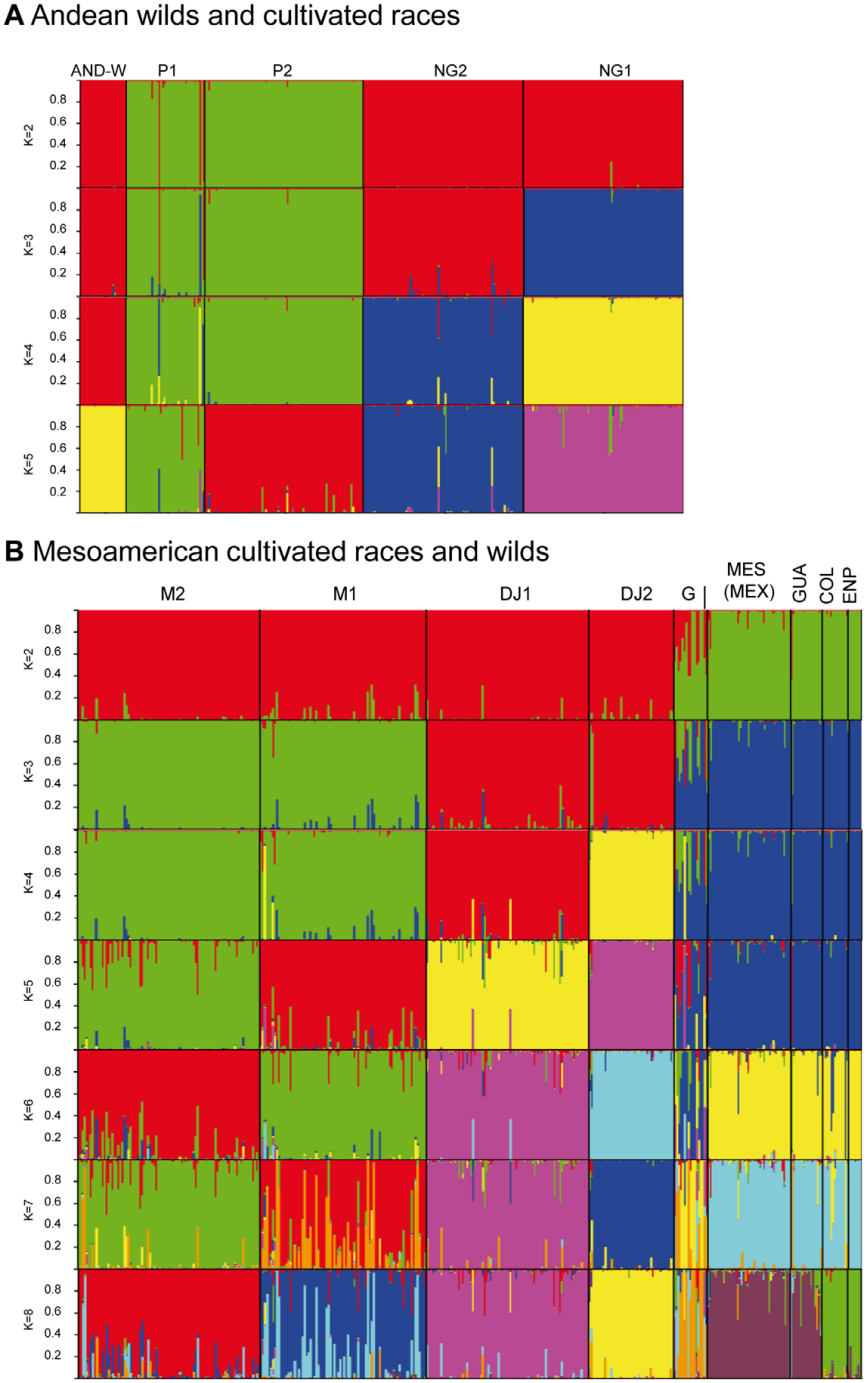
Population structure analysis with the combined dataset of wild and cultivated accessions in A) the Andean genepool and B) the Mesoamerican genepool, with race and sub-population abbreviations as in previous figure.

Separation of the sub-populations and races at each K-value for the STRUCTURE analysis within each genepool was as expected and is presented in Figure 5A and 5B for Andean and Mesoamerican genepools, respectively. In the first part of the figure, the Andean analysis despites K = 2 to K = 5 for Andean wild, race Peru 1 and 2, and race Nueva Granada 1 and 2. Andean wild beans are shown closer to Nueva Granada race especially NG2 than to race Peru, which may reflect the diversity of wild Andean bean genes that may be represented in this race by introgression. In the second part of the figure, the Mesoamerican analysis despites K = 2 to K = 7 for Colombia wild, Guatemala wild, Mexico wild, race Mesoamerica 1 and 2, group Durango-Jalisco 1 and 2 and race Guatemala cultivated. The Mesoamerican cultivated races separate at earlier K values than the wild sub-populations and the Guatemalan subpopulation separated with the Mesoamerican (Mexican) wild beans apart from the Colombian and Northern-Peruvian Ecuadorean sub-populations. Guatemala race presented a high level of admixture and was recognizable as a population precisely because of this behavior.

## Discussion

### SSR Diversity in Wild and Cultivated Beans

Our first major achievement in this study was to determine the SSR alleles found in the largest publically-available collection of wild beans established for diversity assessment and testing, namely the wild bean core collection of 104 genotypes that is part of the FAO collection for *Phaseolus*. The number of wild accessions in bean collections around the world (maximum of 1315 entries in the FAO collection for *P. vulgaris*) is much smaller than the number of cultivated accessions found in such collections (36,000 entries in the FAO collection). As a result the genotypes in the wild bean collection studied here represent between 5 and 10% of the wild beans available to the research community. The wild core collection also has the advantage of having been phenotyped for various agronomic traits, including nutritional quality, aluminum stress tolerance and resistance to several diseases such as angular leaf spot or anthracnose [32].

Another achievement of this study was to find microsatellite markers developed for cultivated bean that worked well in wild bean genotyping (Table 1). Of the 36 markers evaluated by Blair *et al.* [14] for cultivar diversity, all worked well in the fluorescent panels used here for the evaluation of wild bean diversity and all but three markers were single copy across the two sets of genotypes. Therefore, the combined analysis of wild and cultivated accessions from the previous study was straightforward to carry out.

Marker diversity in terms of average number of alleles per marker was high (13) for the combination of 36 markers and 104 genotypes. This surpassed slightly the average of 12 alleles per marker found for 27 SSR markers analyzed in 100 genotypes by [4]: Meanwhile it was slightly lower than the average of 14 alleles per marker found for the same 36 markers in 606 cultivated genotypes in [14]. The similarity of these values is deceptive because the sample size was lower for the wild bean studies compared to the previous study of the cultivated beans [14]. Therefore, diversity in wild beans appears to be somewhat higher than in cultivated beans on a per genotype basis.

### Colombian, Guatemalan and Ecuadorian-northern Peruvian Sub-populations

In terms of population structure, the microsatellites were effective at dividing the wild accessions into five genepools (Figure 1). These included the Andean, Colombian, Ecuadorian-Northern Peruvian, Guatemalan and Mesoamerican genepools. The Andean and Mesoamerican genepools are well-established by various authors who have studied wild bean accessions with various marker types [2], [4], [5], [9] while the three other genepools are more novel. This study confirms that the Andean and Mesoamerican genepools represent the extremes of wild accession diversity. The wild Andean and Mesoamerican beans were the principal primary genepool for domestication events that led to a clear division of all cultivated beans into Andean and Mesoamerican genepools. This division is perhaps the sign of an incipient sub-speciation occurring for wild beans from the two regions at the northern and southern extremes of the species’ geographic range [33].

Among the other wild bean genepools, both the Colombian and Guatemalan genepools were found to be distinct from the Andean and Mesoamerican wild bean genepools. The Ecuadorian-northern Peruvian genepool was related to the Mesoamerican genepool more than the Andean, Colombian or Guatemalan genepools (Figure 1 and 2). The Ecuadorian-northern Peruvian genepool has been proposed as the oldest of the wild bean genepools based on sequencing of the phaseolin gene [33], however Bitocchi *et al.* [15] proposed that this genepool was a relic of an early migration of wild beans to South America. Our results tend to agree with this hypothesis but provide evidence at more widely distributed loci (36 SSRs) than in that previous study (5 gene sequences). While the Colombian genepool was recognized by Rossi *et al.* [5] and Tohme *et al.* [9] based on AFLP diversity studies, the Guatemalan wild bean genepool is proposed here for the first time based on the SSR evaluation and is more specific than the Central American group defined by Kwak and Gepts [4].

The separation of the wild accessions in the neighbor-joining dendogram and in the principal component analysis as well as the distances between wild bean genepools reflected the differences of the sub-populations found in the analysis of population structure. Only a few accessions clustered outside of their assigned genepools in the dendogram and these were for the most part at the base or near the clusters (Figure 2 and 3). The greatest genetic differentiation was between the Ecuadorian-northern Peruvian genepool and the Andean, Colombian and Guatemalan genepool or between the Andean genepool and the Mesoamerican and Colombian genepools (Table 2). This shows that the Ecuadorian-northern Peruvian genepool is unlikely as a progenitor of the full diversity of wild bean as first suggested by Bitocchi *et al.* [15].

Meanwhile, the high F_st_ values we found were similar to the values found by Kwak and Gepts [4] in their differentiation of Mexican, Central American and Colombian wild beans *versus* Ecuadorian-northern Peruvian wild beans. The Mexican genepool in that study corresponds to the Mesoamerican wild beans of this study, a term we use for simplicity as it shows the relationship to Mesoamerican cultivated beans. Rossi *et al.* [5] also uses Mesoamerican as a the name for wild beans from the region and does not recognize a Central American genepool. We suggest that the term Mexican genepool be an alternative for Mesoamerican wild beans.

Meanwhile, the Guatemalan genepool that was novel in the present study was named based on the geographic origin of the wild beans from this mountainous zone of the Neo-tropics. Guatemalan wild beans are known to have a mitochondrial DNA pattern similar to some wild and cultivated Mesoamerican genotypes [34] suggesting their role both in introgression with more northerly South American wild bean populations and with domesticates which may have given rise to the Guatemala race of cultivars [35].

It was also notable in our study that Colombian and Guatemalan genepools were closely related at the K = 3 and K = 4 levels of population structure. Koening *et al.* [7] found that several Colombian genotypes shared the ¨CH¨ phaseolin pattern with wild beans from Guatemala, suggesting gene flow into or from northwest South America from or into Central America at some point in time. Chloroplast DNA analysis of wild accessions by Chacón *et al.* [2] also showed common distribution of haplotypes across South and Central America, that could be explained by isolation by distance and by at least two migration events between Mesoamerica and South America: one from north to south and another one from the region of Colombia to Central America. The geographical isolation of haplotype-defined sub-populations in this previous study was confirmed by results of our study where sub-populations defined by SSR analysis were stratified into specific regions that were divided latitudinally along the Andes Mountains and into Central and North America. Whether this discrete distribution is based on founder effects, geographic isolation by physical barriers or selection for different ecological regions is a matter of interest for evolutionary studies and for understanding bean domestication.

### Inter-genepool Introgression among the Wild Genepools

Our fingerprinting results like those of Rossi *et al.* [5] do suggest bottleneck events for certain wild bean populations from the central and southern Andes where accession diversity was low. However there is equal evidence for introgression between genepools in most areas of northern South America, Central America and Mexico. Geographic isolation was most evident for a set of Argentine wild beans which were genetically very similar and formed the base of the Andean genepool. Similar results were predicted for southern Andean wild beans [36] and for another set of Argentine accessions [37]. Introgression between other groups of wild beans was observed by Tohme *et al.* [9] given that genotypes defined by AFLP markers from these groups had phaseolin alleles from the Mesoamerican genepool. It was also remarkable how divergent the clusters within wild and cultivated common beans were once the main genepool subdivision was considered indicating a large amount of population structure in common beans as a species which agrees with results from other marker studies [4], [14], [15].

A discrete population scenario is useful to understand the structure across multiple cultivated races and nested sub-races, while isolation by distance or a discrete population model with somewhat permeable boundaries is more adequate to characterize the neutral genetic variation of wild common beans. For instance, molecular differences among groups of wild accessions was not as clear as among races in the cultivated types, even though diversity in wild beans appears to be somewhat higher than in cultivated beans.

These contrasting scenarios of population structure in cultivated and wild beans reveal the impact of multiple domestications and divergent human selection on shaping the diversity within cultivars. Therefore, in common bean somewhat like rice [2], several domestications and strong selection processes have given rise to highly differentiated and diverse cultivated genepools and races (Figure 4 and 5). On the other hand, a combination of geographic expansion and contraction of the species and natural selection, may explain variation within wild common beans. In short, distinct demographic and diversification processes may explain why a discrete population scenario is more applicable to describe population structure across cultivars, while a discrete population model with somewhat permeable boundaries is more adequate to understand variation of wild common beans.

### Additional Questions and Conclusions

Other interesting questions remain. The intermediate position of the cultivated Guatemala race (Figure 5) may suggest it as an introgression bridge between Mesoamerican wild sub-populations and cultivated races with Northern South American populations. However, it also may be regarded as a third independent domestication event. Further study is needed on whether introgression was symmetric or asymmetric between genepools and races, or whether it was actually an artifact of extensive ancestral polymorphism. Finally, we were unable to pin-point where the multiple and independent domestications occurred. The answer may be that there were more domestications and introgression events than initially thought, and that they were carried out in a stepwise manner at different times and locations in various societies of the Americas rather than as unique events in single locations.

We can conclude that the wild Andean and Mesoamerican genepools were the principal sources of domesticates which were probably enhanced by introgression from some of the other three wild genepools (Colombian, Ecuadorian-northern Peruvian and Guatemalan). This introgression would have helped to create the race structure observable today in cultivated common beans, especially for race Nueva Granada in the Andean genepool and race Guatemala in the Mesoamerican genepool. Finally, fingerprinting with fluorescently-labeled microsatellites was an excellent technique for the evaluation of wild bean populations, for studying population structure and dynamics, as well as for linking wild sub-populations to cultivar races. Some evidence was found for a bottlenecks that might have occurred in the derivation of the Andean genepool from Mesoamerican ancestors but less upon domestication of the crop by New World farmers when enhanced diversity was selected along the road to the creation of bean races. The wild bean core collection will be valuable for further phenotyping, diversity assessment or association mapping and is proposed as a starting point for studies of wild accessions of this species [38], [39]. Therefore, this constitutes an ideal system to study diversification, domestication and adaptive processes across two of the most diverse hotspots for genetic resources in the world: the Andes and Mesoamerica.

## Supporting Information

Figure S1 Natural logarithm of the likelihood and Evano’s delta K for the structure analysis conducted for wild accessions, for Andean cultivated and wild accessions, for Mesoamerican cultivated and wild accessions, and for all cultivated and wild accessions.
(PSD)

Table S1 Wild and cultivated accessions used in this study depicting the region/country where they were collected and the genepool to which they were assigned.
(XLSX)

Table S2 AMOVA details for the wild analysis.
(XLSX)
